# Clinical Multi-Omics Study on the Gut Microbiota in Critically Ill Patients After Cardiovascular Surgery Combined With Cardiopulmonary Bypass With or Without Sepsis (MUL-GM-CSCPB Study): A Prospective Study Protocol

**DOI:** 10.3389/fmed.2020.00269

**Published:** 2020-07-08

**Authors:** Wenyan Ding, Jianzhou Liu, Xiang Zhou, Qi Miao, Haibo Zheng, Baojin Zhou, Guifang Dou, Yigang Tong, Yun Long, Longxiang Su

**Affiliations:** ^1^Department of Critical Care Medicine, Peking Union Medical College Hospital, Peking Union Medical College, Chinese Academy of Medical Sciences, Beijing, China; ^2^Department of Cardiac Surgery, Peking Union Medical College Hospital, Peking Union Medical College, Chinese Academy of Medical Sciences, Beijing, China; ^3^Deepxomics Co., Ltd., Shenzhen, China; ^4^Department of Pharmaceutical Sciences, Beijing Institute of Radiation Medicine, Beijing, China; ^5^Beijing Advanced Innovation Center for Soft Matter Science and Engineering (BAIC-SM) College of Life Science and Technology, Beijing University of Chemical Technology, Beijing, China

**Keywords:** cardiac surgery, gastrointestinal microbiome, metagenomics, metabolomics, sepsis

## Abstract

**Introduction:** Fever of unknown origin (FUO) and hemodynamic instability are complications that develop after cardiac surgery combined with cardiopulmonary bypass (CPB) for heart disease. Patients who develop fever with hemodynamic instability after cardiac surgery may have systemic inflammatory response syndrome or sepsis. Cardiopulmonary bypass (CPB) is a technique that temporarily takes over the function of the heart and lungs during cardiac surgery. Recent reports suggest that early bloodstream infections of patients undergoing CPB are due to gram-negative bacteria that are present in the intestinal flora. The theory of intestinal flora translocation has growing evidence. Intestinal ischemia-reperfusion that occurs during cardiac surgery with CPB will induce a systemic inflammatory reaction and may cause intestinal flora translocation. Does this systemic reaction cause sepsis? We therefore propose this protocol to determine whether the changes in the intestinal flora in patients after cardiac surgery with CPB are related to sepsis.

**Methods and Analysis:** This study is a prospective observational case–control study to analyze the variation in the intestinal microflora and metabolites in patients undergoing cardiac surgery with CPB and to observe the outcomes of patients with routine clinical interventions. The control group will include healthy people without intestinal illness. Feces and blood samples will be acquired 1 day before cardiac surgery and within 24–72 h after cardiac surgery, and will be used for genomics and metabolomics analyses. Demographic data describing age, sex, main diagnosis, and past medical history and data related to the CPB time and application of antibiotics are available. Sequential (sepsis-related) organ failure assessment, infection-related laboratory items, infection site, and pathogenic microorganisms, and nutrition, and gastrointestinal function assessment are additionally recorded. Group analysis of data will be conducted according to the outcomes (sepsis vs. non-sepsis and survivors vs. non-survivors).

**Ethics and Dissemination:** This protocol has been ethically approved by the Ethics Committee of Peking Union Medical College (ID: ZS-1612). Informed consent will be obtained before subject enrolment, and data will be stored in a secured database. The results will be submitted to international peer-reviewed journals and presented at international conferences.

**Trial Registration Number:** NCT04032938.

## Introduction

Complications after cardiac surgery are important factors that increase postoperative mortality after surgery (1). A prospective cohort study involving 4,446 consecutive patients undergoing cardiac surgery showed that there are some critical postoperative complications after cardiac surgery, including infection, renal failure, pulmonary complications, gastrointestinal complications, and multiorgan failure (2). The results showed that the in-hospital mortality was 3%, the postoperative renal failure incidence was 10%, the pulmonary complication rate was 32%, the gastrointestinal complication rate was 8%, the neurological complication rate was 7%, and the multiorgan failure rate was 2%. After undergoing cardiac surgery, patients are usually critically ill and require intensive care. Interestingly, we know from experience that some patients develop fever and hemodynamic instability after cardiac surgery without definitive etiological evidence. Previous studies have shown that systemic inflammatory response syndrome and postoperative fever are complications that develop after cardiac surgery (3, 4). We speculate that fever of unknown origin (FUO) and hemodynamic instability are related to systemic inflammatory response syndrome or sepsis. However, they may not be. The use of CPB or extracorporeal life support (ECLS) to support a patient during an intraoperative thoracic surgery is common, and cardiopulmonary bypass (CPB) is one of the risk factors for infection in patients undergoing cardiac surgery (5). On the one hand, it is reported that most of the early bloodstream infections of patients undergoing CPB are caused by gram-negative bacteria, which are commonly present in the intestinal flora (6). This finding indicates a relationship between the intestinal flora and infection or sepsis. On the other hand, the CPB process and duration of CPB determine the severity of organ ischemic injury. Intestines, as an ischemia-sensitive organ, will allow translocation of bacteria and endotoxins to the bloodstream (7). Therefore, the intestines play a role in postoperative sepsis or infection.

There is growing evidence that microbiomes play an indispensable role in human health and disease. Meanwhile, a lot of progress has also been made in association studies between disease and intestinal metabolites. As early as the later part of the nineteenth century, researchers found that peritonitis could result from the passage of bacteria from organs adjacent to the peritoneal cavity. The “gut origin of sepsis” became a theory of interest to clinicians (8). The theory of intestinal flora translocation has gradually emerged. Dickson et al. found the lung bacteria of patients with ARDS, which is correlated with systemic inflammation translocated from the intestine (9). Singer et al.'s research in mice and patients who died of sepsis suggests that bacterial translocation could be associated with sepsis in acute neuroinflammation (10). Other studies found that a ratio of Bacteroidetes to Firmicutes (B/F ratio) of >10 or <0.10 in the gut microbiota may suggest a high risk of death (11). We hypothesized that, except for iatrogenic infections, endogenous bacterial translocation after CPB is a possible mechanism of early sepsis or septic shock in these patients. The use of intestinal microecological studies and metabolite studies can help determine whether changes in the intestinal flora and metabolites after cardiac surgery with CPB are associated with sepsis. This research method has become very mature in clinical research. There are a series of studies on the host microbiota with neurologic, metabolic, cardiovascular, or gastrointestinal disorders (12–16). Studies on the association between disease and intestinal metabolites have also made essential progress. We hypothesize that sufficient information related to the pathogenesis of sepsis can be obtained from studies on intestinal microecology and metabolites.

This study will identify the variation in the gut microbiota and host metabolite profiles of patients after undergoing cardiac surgery with CPB. The changes in the intestinal flora in patients with sepsis and patients with worse outcomes will be analyzed emphatically. Combined with clinical data, the possible pathogenesis of FUO and patients with hemodynamic instability and their association with infection will be analyzed. This study may also indicate whether the phenomena of FUO and hemodynamic instability are attributed to sepsis or a purely inflammatory response.

## Method and Analysis

### Study Design

**Figure 1:**
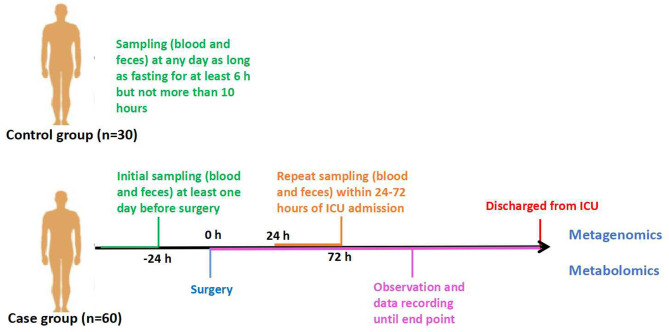
Study design.

This protocol is designed as a prospective observational case–control study in patients who underwent cardiopulmonary bypass (CPB) due to cardiac surgery. Thirty healthy persons aged between 18 and 85, without digestive system disease, will be selected as the control group. The case group will include patients admitted to the intensive care unit (ICU) after cardiac surgery with CPB, which is performed by the Department of Cardiac Surgery of Peking Union Medical College Hospital. The patients enrolled should be divided into two groups according to their outcomes: one group of non-sepsis patients and the other group of sepsis patients after cardiac surgery. Sample collection will be terminated when each group receives 30 cases. These 60 cases will be regarded as the case group. Additionally, all the patients we observe will be further divided into survivors and non-survivors based on 28-days survival. Feces and blood samples will be obtained at certain time points (Figure 1).

The fecal sample analysis will apply metagenomics, and the feces and blood samples will be analyzed using an untargeted metabolomics method. We intend to explore the stratification of gut microbial communities in patients who underwent cardiac surgery with CPB and to analyze the variation in metabolites in patients' plasma and fecal samples. Predictive biomarkers and possible pathogenesis of FUO and hemodynamic instability after CPB will also be provided by clinical outcome analysis combined with a multi-omics study.

### Study Population

The inclusion criteria are the following. Patients between 18 and 85 years of age who will undergo extracorporeal circulation during cardiac surgery, will be admitted to the ICU and provide written informed consent. The control group will be 30 healthy persons aged between 18 and 85 without digestive disease. The exclusion criteria are as follows; patients would be excluded if they (1) had a fever before surgery, regardless of the etiological evidence of infection; (2) had anti-infective treatment before surgery; (3) had gastrointestinal surgery that left the digestive system dysfunctional; (4) had a history of CPB in the last 6 months; or (5) rejected or abandoned ICU therapeutic intervention. The control group will be excluded if they are taking certain medications.

### End Point

The observation and sample collection will be terminated after 28 days after surgery or for reasons of (1) death or if (2) the patient is satisfied with the indications for discharge from the ICU.

### Study Protocol

#### Recruitment

Patients in the department of cardiac surgery who underwent cardiac surgery with cardiopulmonary bypass will be assessed for eligibility for the study according to the inclusion and exclusion criteria. Additionally, the control group will be recruited from people who will have health checks at Peking Union Medical College Hospital or the healthy volunteers. When people agree to participate, the informed consent forms will be signed in duplicate. Recruitment will be terminated when the case group reaches 60 cases. The study protocol was approved by the Ethics Committee. The committee will oversee the study to monitor data safety. The collection started on August 1, 2018 and is expected to finish on September 30, 2020. We have collected a total of 67 samples, including 45 cases of cardiac surgery patients in the case group and 22 healthy people in the control group have been collected on January 31, 2020.

#### Sample Collection

There will be 60 patients enrolled as the case group and 30 healthy people as the control. After fasting for at least 6 h but not more than 10 h, venous blood will be collected by direct venepuncture or established venous access from patients enrolled. Plasma will be taken from the blood and remain in Eppendorf tubes at −80°C for 1 h. Fresh fecal samples will be obtained (sometimes aided by glycerinum) and stored at −80°C before analysis. In the control group, samples will be collected once. In the case group, feces and blood samples will be acquired at the following time points (Figure 1): (1) T1: 1 day before cardiac operation; and (2) T2: within 24–72 h of ICU admission, sampling must be performed before patients start oral feeding.

#### Study Setting

This study is an open-label, single-center, prospective, observational study at one center in China.

#### Observation and Data Recording

Demographic data describing age, sex, main diagnosis, and past medical history are available at the inception of the study. Data related to the CPB time and application of antibiotics post-operation are available on the MUL-GM-CSCPB study Data Recording Sheet (Supplementary Table). The following observational data will be measured and recorded when patients are admitted to the ICU and at every sampling time.

1. Sequential (sepsis-related) organ failure assessment includes blood pressure; mean arterial pressure (MAP); and vasoactive drugs, including noradrenaline (NE), epinephrine (E), and dopamine (Dopa); PaO_2_/FIO_2_; Glasgow Coma Scale score; creatinine or urine output; bilirubin; and platelet count.
2. Infection-related items include body temperature, WBC count, neutrophils, PCT, and antibiotics.
3. Whether to be diagnosed with sepsis (according to Sepsis 3.0).
4. Infection site and pathogenic microorganism.
5. Nutrition and gastrointestinal function assessment including enteral or parenteral nutrition, bowel dilatation tested by ultrasound, gastric residual volume and intra-abdominal pressure measured via bladder.

#### Grouping by Outcomes

The primary outcome is patients with cardiovascular surgery with CPB and healthy people.

The secondary outcomes are as follows.

After sample collection, the case group will be selected and divided into two groups according to their clinical outcomes.

1. Sepsis group (sub grouped by suspected or documented infection) and non-sepsis group;
2. Survivors and non-survivors based on the 28-days survival.

#### Definition

The clinical criteria according to the third international consensus (Sepsis-3) (17):

Sepsis: suspected or documented infection and an acute increase of ≥2 SOFA points.

Suspected infection group will be identified as those who had body fluids sampled for culture and received antibiotics but had a negative culture result.

The documented infection group will be identified as those who had body fluids sampled for culture with a positive result.

Survivors: Patients that survive for 28 days or more after surgery.

Non-survivors: Patients that survive <28 days after surgery.

#### Protocol Feasibility

We will undertake a preliminary study in our center before conducting the full-scale research project. It will take at least 3–4 months (including an enrolment period of about 3 months) to recruit ~15 patients and 15 healthy people as controls. We aim to address current uncertainties regarding the feasibility of sampling and parameters. If at least 80% of patients have valid and complete data of the MUL-GM-CSCPB study Data Recording Sheet (Supplementary Table), the pilot study will be successful and data from the pilot phase will be included into the final analysis. Some modifications will be made in the protocol if it is hard to reach the criteria. Data from the pilot phase will be included into the final analysis if no modification has been made.

### Experimental Analysis

#### Metagenomic Analysis

We chose metagenomics for sequencing studies of intestinal microecology. The metagenomics data will be annotated by comparing the predicted genes with the non-redundant protein sequence (NR) database. The metagenomic annotations provide comprehensive species information, including not only bacteria but also fungi, archaea, and viruses. First, DNA extraction will be performed using commercial kits according to the manufacturers' instructions. Libraries will be constructed according to a commercial kit and sequenced using the paired-end method and 93 cycles of sequencing. After read processing, we will perform taxonomic annotation according to related software. We will search every identified sequence from the UniProt/Swiss-Prot database (18) and subsequently retrieve the protein family, KEGG orthologous group and pathway information (19) associated with each UniProt/Swiss-Prot accession number. The relative proportion of read counts will be used as a quantitative estimation of the abundance of each taxon or function. We will keep the metagenomic sequence data on an open access site.

#### Metabolomics Analysis

The blood and stool samples will be analyzed using untargeted metabolomics. The analysis will be performed on an ultra-high-performance liquid chromatography system. To detect as many metabolites as possible, we plan to perform both polar ionic and lipid modes depending on the properties of the serum metabolites. The raw data will be converted into mzXML format and then processed by XCMS (20) software for peak picking and alignment. Metabolite features generated from XCMS will be further quantified by metaX software (21), including missing value imputation, data filtering, normalization, and comparison. Significant metabolites will be calculated using univariate (T/Wilcoxon test) and multivariate (PCA and PLS-DA) analyses integrated in metaX. The online HMDB database (22) and KEGG database (19) will be used to annotate the polar ionic metabolites by matching the exact molecular mass. The MS/MS spectrum from HMDB, MassBank (23) and an in-house database will assist in metabolite identification. For lipid identification, a LipidBlast database (24) will be used by matching the MS/MS spectrum and retention time. MetaboAnalyst (25) will be used for the identification of metabolic pathways.

### Multi-Omic Data Analysis Plan

The data will be analyzed according to the case and control groups, sepsis and non-sepsis groups, and survivor and non-survivor groups, or some other classifications based on the pathophysiology.

Metagenomic and metabolomics statistical analysis will be performed using commercial software. To account for dependencies in repeatedly measured observations within a subject, a linear mixed-effect model will be used to assess the differences between the case group and the control group. Statistical analysis will be performed using SPSS (version 22, SPSS, Chicago, IL). A two-sided significance level of 0.05 will be used for statistical inferences. Combining principal component analysis (PCA), partial least squares discriminant analysis (PLS-DA), and orthogonal partial least square discriminant analysis (OPLS-DA), different metabolite profiles will be identified.

Receiver operating characteristic (ROC) analysis will be conducted to evaluate the utility of the potential biomarkers selected. Multivariate logistic regression analysis will be used to show the correlation between each factor and prognosis and changes in intestinal flora. A *p* < 0.05 will be considered statistically significant.

## Discussion

This study is a prospective observational case–control study to analyze the variation in the intestinal microflora and metabolites in patients undergoing cardiac surgery with CPB and to observe the outcomes of patients with routine clinical interventions. Sixty patients will be enrolled to observe their outcomes (sepsis or non-sepsis) and their survival outcomes (28-days survival or death). Another 30 healthy people will be enrolled as the control group. We intend to collect blood and feces from these persons and to perform genomic and metabolome tests on pre- and post-surgery samples or control samples. According to bioinformatics analysis combined with mathematical modeling methods and statistical analysis, the relationship between intestinal microecological changes and sepsis will be determined.

We believe that FUO with hemodynamic instability after surgery is commonly seen and is likely to be a manifestation of the inflammatory response with sepsis. However, accurate diagnosis is quite difficult due to the low rate of detection of etiology. Infectious complications, especially sepsis or septic shock after cardiopulmonary bypass (CPB), are known to be a critical issue associated with severe morbidity and mortality (26). We know that intestinal ischemia-reperfusion occurring during cardiac surgery with cardiopulmonary bypass (CPB) will induce a systemic inflammatory reaction. Prolonged CPB could increase intestinal permeability and thus lead to endotoxin or bacterial translocation from the intestine to the bloodstream (27). Researchers determined intestinal fatty acid-binding protein (I-FABP), tumor necrosis factor alpha (TNF-alpha), interleukin 6 (IL-6), interleukin 8 (IL-8), and endotoxin levels in arterial blood at different times of CPB to indicate that the release of biomarkers that indicate ischemia-reperfusion damage to the gastrointestinal mucosa and endotoxemia may identify intestinal damage and bacterial translocation.

The current view suggests that intestinal bacteria may lead to sepsis in several ways. First, intestinal bacteria and bacterial products are transferred to distant organs through blood flow. However, this view lacks bacteraemic evidence. Second, intestinal bacterial metabolic products are transferred to distant organs through the lymphatic pipeline. Third, in critically ill patients, changes in the intestinal flora and metabolites can cause systemic inflammatory reactions and the body's immune response, leading to sepsis (26). We suspect that intestinal dysbacteriosis after CPB is a possible mechanism of inflammation and anti-inflammatory immune imbalance, and that bacterial translocation leads to infection and sepsis. In the critical care unit, most common clinical interventions [e.g., enteral feeding (28), proton-pump inhibitors (29), systemic catecholamines (28), and systemic antibiotics (30)] will change intestinal bacteria. It was recently shown that exposure to broader-spectrum antibiotics during hospitalization is associated with dose-dependent increases in the risk of subsequent sepsis (31). Moreover, in critically ill patients, a significant decrease in bacterial diversity was observed. In more than 30% of patients, a single bacterial genus makes up >50% of the gut microbiota (32). Furthermore, in many cases of prolonged critical illness, only two-member pathogen communities remain. The communities contain bacteria associated with the genera Enterococcus and Staphylococcus, and the family Enterobacteriaceae comprises the majority of these communities (33).

Several studies have shown that the role of inflammation in heart failure may promote the gut microbiota as a cardio-metabolic target for intervention (34). The gut microbiota can secrete large amounts of amyloids and lipopolysaccharides, which might produce some proinflammatory cytokines, such as tumor necrosis factor (TNF) (35). TNF could reduce mitochondrial activity, alter calcium homeostasis, and impair β-adrenergic signaling in cardiomyocytes to act as a suppressor of cardiac function (36). It can be seen that the study of intestinal microecology is of great significance for the postoperative inflammation response or sepsis and cardiac function. Therefore, we are reasonably confident that intestinal flora research can be an important breakthrough in the study of the pathogenesis of postoperative sepsis. Differences in species of the intestinal flora also affect the prognosis of sepsis.

A normally functioning intestinal mucosa prevents the transfer of enteric bacteria and endotoxins into other organs and blood circulation. The gut barrier is disrupted when patients suffer from impaired intestinal blood supply and insufficient nutrient support (37). Intestinal barrier function is composed of mucosal immunity and physical integrity. In a review article, antioxidant treatment displayed beneficial effects on intestinal barrier integrity and significantly reduced the incidence of bacterial translocation (38). Therefore, we can conclude that nutrition is likely to be an effective treatment to improve immunity and repair the intestinal barrier. How can this clinical problem be addressed? We hypothesize that in critically ill patients who require nutritional support, the current guidelines recommend the use of enteral nutrition within 24–48 h and advance toward optimal nutritional goals over the next 48–72 h. The absence of enteral nutrition will deregulate the receptors that modulate the immunological response and initiate intestinal inflammation. Studies show that the physiological stimulus of enteral nutrition is crucial to maintain gastrointestinal functions such as barrier and immunological function (39). Treatment in some studies with microbiota-targeted metabolites, such as butyrate or other short-chain fatty acids (SCFAs), could be used as modulators (40). Fiber supplementation of feeds has the potential to improve microbiota mass and function and produce SCFAs to activate the colonic microbiota (41). Modulation of the microbiota could be achieved by administration of probiotics and dietary interventions to supply beneficial microbes (42). Administrating probiotics, improving the intestinal microenvironment by dietary and prebiotics, and recolonizing the gut with fecal microbiota transplantation (FMT) could be pathways in the management of sepsis (43). Studies on nutrition and the intestinal barrier will be an advanced and promising research direction in the future.

## Conclusion

This study will provide the most comprehensive description to date of host and microbial activities in patients who underwent CPB. To investigate the features of intestinal microecology and intestinal and blood metabolites in patients who underwent cardiovascular surgery with CPB, it is of great significance to study the specific relationship and potential mechanism of FUO with hemodynamic instability after CPB.

## Data Availability Statement

The raw data supporting the conclusions of this article will be made available by the authors, without undue reservation.

## Ethics Statement

The study protocol was approved by the institutional review board at the participating center. Data will be presented at international conferences and published in peer-reviewed journals.

## Author Contributions

LS and YL contributed to the proposal and design of the study protocol. XZ made some key recommendations for the study design. WD, JL, and LS wrote the study protocol and designed the CRF chart used in this study. JL, HZ, and QM were responsible for sample collection. BZ is responsible for statistical and bioinformatic guidance. GD contributed to the metabolomics analysis. YT provided advice for metagenomics analysis. All authors critically reviewed the manuscript.

## Conflict of Interest

BZ is an employee of Deepxomics Co., Ltd. The remaining authors declare that the research was conducted in the absence of any commercial or financial relationships that could be construed as a potential conflict of interest.
