# Supplementary material for: Clinical Multi-Omics Study on the Gut Microbiota in Critically Ill Patients After Cardiovascular Surgery Combined With Cardiopulmonary Bypass With or Without Sepsis (MUL-GM-CSCPB Study): A Prospective Study Protocol
Source: Front Med (Lausanne). 2020 Jul 8;7:269. doi: 10.3389/fmed.2020.00269 (PMC7360671; doi:10.3389/fmed.2020.00269)
Supplement: Supplementary file 1 [file Table_1.pdf]

MUL-GM-CSCPB Study

Data Recording Sheet

**Full name**		**ID**		**Sex**		**Age**		**Ad-ICU date**		**Dis-ICU date**	
**Diagnosis**						**Cardiopulmonary bypass time(min)**					
**Infection source**				**Primary Outcome**		1. Non-sepsis<br>**2.** Sepsis			**Survival time (d)**		
**SOFA (if infection)**			**APACHⅡ**			**Secondary Outcome**	1.ICU Survival		2. Non-survival		
**Past medical history**	hypertension; diabetes; COPD; chronic heart disease; chronic kidney disease; immune suppression; nervous system disorders; blood diseases;										
**Primary antibiotics post-operation**											

	**Before Cardiac Surgery**	**Post-operative (in 24-72h)**	Variables (worst value)	**Post-operative**
			Body temp, °C (worst – high or low)	
			WBC count (1000/cu mm)	
			Neutrophils (%)	
			PCT (ng/mL)	
			antibiotics	
			Is it sepsis?	
			pathogenic microorganism(name)	
			Infection site	
			Nutrition (1. Enteral; 2. Parenteral;)	

			bowel dilatation	
			gastric residual volume	
			Intra-abdominal pressure (IAP)	
